# Evolution of isolated systolic hypertension with normal central blood pressure in adolescents—prospective study

**DOI:** 10.1007/s00467-020-04731-z

**Published:** 2020-09-03

**Authors:** Łukasz Obrycki, Janusz Feber, Grażyna Brzezińska, Mieczysław Litwin

**Affiliations:** 1 Department of Nephrology, Kidney Transplantation and Hypertension, The Children’s Memorial Health Institute, Aleja Dzieci Polskich 20, 04-730 Warsaw, Poland; 2 Division of Nephrology, Department of Pediatrics, The Children’s Hospital of Eastern Ontario, Ottawa, Canada; 3 Department of Cardiology, The Children’s Memorial Health Institute, Warsaw, Poland

**Keywords:** Isolated systolic hypertension, Children, Spurious hypertension, Central systolic blood pressure, Uric acid, ABPM

## Abstract

**Background:**

The clinical significance of isolated systolic hypertension with normal central blood pressure known as spurious hypertension (sHT) in adolescents and its evolution over time is not known.

**Methods:**

The aim of this study was to analyze changes in office, ambulatory blood pressure (ABPM), central systolic blood pressure (cSBP), hemodynamic parameters, and target organ damage (TOD) over a 1-year follow-up in a group of non-obese children with sHT.

**Results:**

Of 294 patients referred for primary hypertension, 138 patients (31 girls; 22%) had hypertension confirmed by ABPM. 48/138 (35%) patients (7 girls; 15%) were diagnosed with sHT (elevated office and ambulatory systolic BP, but normal cSBP); 43 of them (6 girls; 14%) were followed for 12 ± 3 months during non-pharmacological therapy. At baseline 7 (16%) patients had borderline values of cIMT or LVMi indicating mild TOD. After 12 months, 10/43 (3 girls; 23%) patients developed sustained HT (elevated office, ambulatory BP and cSBP), 11/43 (1 girl; 26%) maintained sHT, and 22/43 (2 girls; 51%) evolved to white coat hypertension or normotension. The cSBP values increased in 27 patients (4 girls; 63%), but the group average remained in the normal range. Prevalence of TOD did not change during observation. The multivariate regression analysis showed that the only predictor of cSBP change over time was a change in serum uric acid level.

**Conclusions:**

In conclusion, after 1 year of non-pharmacological treatment, 23% of adolescents with sHT developed sustained hypertension, with the main predictor of cSBP change being the change in serum uric acid.

**Electronic supplementary material:**

The online version of this article (10.1007/s00467-020-04731-z) contains supplementary material, which is available to authorized users.

## Introduction

Spurious hypertension (sHT) is a phenotype of primary hypertension (PH) defined as elevated office and ambulatory systolic blood pressure (SBP), yet maintaining normal central systolic blood pressure (cSBP) and presents clinically as isolated systolic hypertension (ISH). sHT was originally described by O’Rourke et al. in a group of 6 adolescent and young adult males aged 14–23 years who all had office ISH with normal cSBP, none of whom presented with hypertensive target organ damage (TOD) [1]. In most reports sHT was described mainly among males. The more recent large prospective Chicago Heart Association Detection Project in Industry Study with 30 years of follow-up demonstrated that adult males but not females with ISH (cSBP was not assessed) had a low risk of cardiovascular events and cardiovascular death, compared with subjects with high-normal blood pressure (BP) [2].

Because ISH is the dominant hemodynamic phenotype in children with PH and males dominate among hypertensive children, it is important to assess the risk associated with ISH and normal cSBP in youth with PH.

To date, only a few studies have assessed the prevalence of sHT among adults with ISH and its clinical relevance. Studies by Saladini et al. and Palatini et al. showed that sHT in young adult males is a mild condition and patients with sHT are at a low risk of developing sustained hypertension (15.2%), similar to the normotensive population (14.7%). Thus, it was proposed that subjects with sHT and low cardiovascular risk do not require extensive diagnostics and pharmacological treatment [3, 4]. The importance of cSBP was documented in adult studies showing that cSBP better correlated with TOD than with peripheral BP [5–8].

Although ISH is the predominant hemodynamic phenotype of PH in children and young adults, there are only single reports on the prevalence and association of sHT and TOD among adolescents. Previously we showed that 35% of children with office and ambulatory (ABPM) hypertension had brachial systolic hypertension with normal cSBP, i.e., fulfilled criteria of sHT and had a lower risk of TOD [9]. They also had lower body mass index (BMI) and waist circumference (WC) than those with “true” hypertension, i.e., both with elevated brachial and central blood pressure. It was also found that cSBP positively correlated with left ventricular mass index (LVMi), carotid intima-media thickness (cIMT) and pulse wave velocity (PWV). cSBP also had a greater predictive power than 24-h SBP in predicting LVH [9]. This suggests that adolescents with sHT may be at a lower risk of developing TOD compared with patients with both elevated brachial and central blood pressure. However, there is no prospective data on the evolution of sHT in adolescents and the risk of development of TOD later in life.

The aim of our study was to analyze the longitudinal changes of office, 24-h ABPM, cSBP, hemodynamic and TOD parameters over a 1-year follow-up period in a group of children with sHT.

## Methods

This study was approved by the Bioethical Commission of the Children’s Memorial Health Institute. All patients and their parents gave informed consent. The study meets the criteria of the 1975 Declaration of Helsinki revised in 2013. The study subjects were recruited from a cross-sectional, previously reported study [9]. All consecutive patients who were referred in years 2015–2019 because of arterial hypertension and in whom ultimately PH was diagnosed were included in this study. All patients underwent full diagnostic evaluation according both to local and the pediatric guidelines of the European Society of Hypertension, including anthropometric measurements and biochemical tests, assessment of office blood pressure, 24-h ABPM, hemodynamics, echocardiography, cIMT, PWV, and pulse wave analysis (PWA); secondary causes of arterial hypertension were excluded [10–12].

**Fig. 1:**
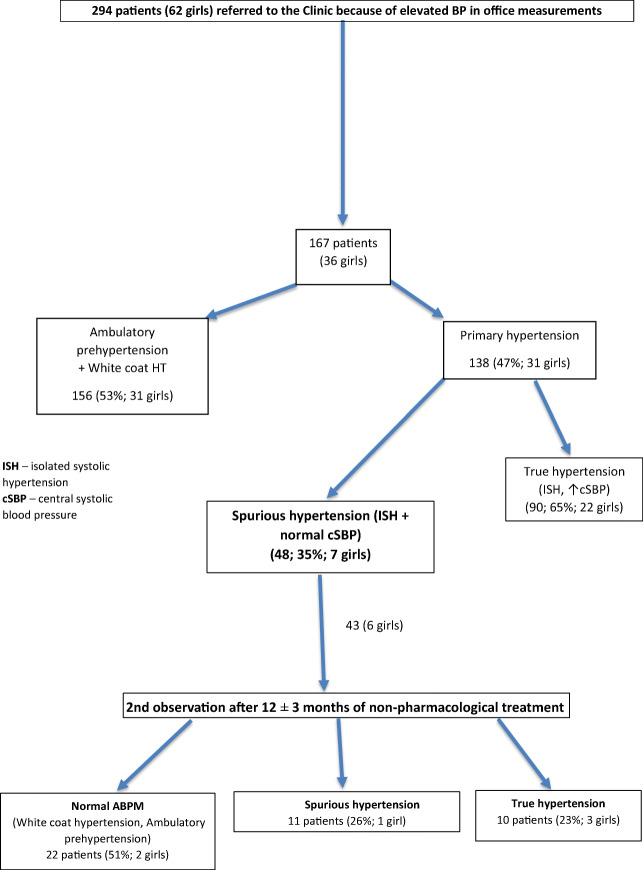
Scheme of the study. ISH, isolated systolic hypertension; cSBP, central systolic blood pressure; TOD, target organ damage; cIMT, carotid intima-media thickness; PWV, pulse wave velocity; LVH, left ventricular hypertrophy

At baseline, out of a total of 294 consecutively referred patients with PH, 127 (43%) patients were diagnosed with white coat hypertension (WCH) and 29 (10%) patients with ambulatory prehypertension (AmbPreHT). In the group of patients with both office and 24-h ambulatory hypertension (*n* = 138; 31 girls; 22%), 48 (35%) patients (7 girls; 15%) presented with sHT phenotype (office and ambulatory ISH, but normal cSBP) and 90 (65%) patients had true hypertension (tHT; elevated office, ambulatory and cSBP). Out of 48 patients with sHT, 43 (6 girls; 14%) with median age 16.7 (8.5–17) years were followed for 12 ± 3 months during non-pharmacological therapy and were included in the current study; 5 were lost to follow-up (Fig. 1). Non-pharmacological therapy was based on uniform dietary advice and prescription of moderate to vigorous physical activity for at least 60 to 90 min daily.

### ABPM

All ABPM measurements were assessed oscillometrically by means of the SpaceLabs Monitor 90,207, using the most appropriate cuff fitted to the non-dominant arm. Readings were taken every 20 min during the day and every 30 min at night. Recordings lasting ≥ 20 h with ≥ 80% of readings were considered valid and were included in the analysis. Patients completed a diary for the identification of active and sleep periods. We used a recent classification system based on ABPM [10]. In children > 16 years of age, the adult cut-offs were used if obtained BP values exceeded criteria for adults [11].

### PWV and PWA measurements

PWV and PWA were measured non-invasively with the oscillometric method using a Vicorder® (SMT Medical) system device. It has been validated against applanation tonometry systems (Sphygmocor®) and invasive measurements of cSBP and was found to be a reliable and simple alternative to tonometry [13, 14]. Moreover, cSBP by Vicorder was more closely related to non-invasive measurements than tonometry measurements. This method is investigator-independent and is recommended in studies of large groups of subjects [15]. Vicorder® has also been validated in pediatric studies [14]. Because normative pediatric data obtained with oscillometric device used the 97th percentile as the upper limit of normal PWV, we also considered standard deviation score (SDS) > 1.88 (> 97th percentile) as a cut-off value for an elevated PWV [16].

PWA enables calculation of parameters describing the characteristics of the arterial system including cSBP, augmentation pressure (AugPress), augmentation index (AugInd), central pulse pressure (cPP), cardiac output (CO), cardiac index (CI) and total peripheral resistance (TPR). PWA and cSBP assessed by oscillometric measurements with Vicorder were validated against SphygmoCor and obtained results did not differ between the two methods [13]. We used pediatric normative values for cSBP obtained with an oscillometric device (Mobil-O-Graph, I.E.M., Stolberg, Germany) [17]. Measurement was performed in the supine position after 5 min of rest, according to published guidelines [16].

### Echocardiography

All measurements were performed according to the American Society of Echocardiography guidelines. To standardize left ventricular mass to height, LVMi was calculated according to the de Simone formula [18, 19]. LVH was defined as an LVMi value above the 95th percentile for age- and sex-based reference data [20]. Significant LVH was defined as LVMi ≥ 51 g/m height^2.7^.

### cIMT measurements

The cIMT measurements were performed using the Aloca Prosound Alpha-7 machine (5.5–12.5 MHz) in accordance with Mannheim Consensus recommendations. The obtained results were averaged independently for the right and left common carotid arteries; the average value for both sides was then converted into SDS based on pediatric normative values [21]. Values of cIMT above 1.65 SDS were considered abnormal (above 95th percentile) [10, 22–24].

### Laboratory Investigations

The blood used for assessment of plasma glucose levels, lipid profile and serum uric acid (UA) levels, was taken after 12 h of fasting. We used unit c501 as a biochemistry analyzer for spectrophotometric, immunoturbidimetric and ion-selective determination of the above biochemical parameters.

## Longitudinal assessment

All the tests were performed at baseline and after 12 ± 3 months.

## Statistical analyses

Calculation of power size to detect change in effect size of 0.5 in longitudinal analysis in paired test showed 89% power in paired test for 43 patients. However, it was lower for comparison 10 vs. 33 patients in unpaired test. Thus, additional tests (Cohen effect size) were done to improve sensitivity of statistical analysis.

cSBP and 24-h ABPM were expressed in absolute values as mmHg. We also calculated the index of the patient’s 24-h SBP and DBP value to the upper limit of normal (95th percentile); an index > 1.0 represents abnormal/elevated values in relation to the 95th percentile of normal values. Twenty-four-hour mean arterial pressure (MAP) values were presented as absolute (mmHg) and standard deviation score (SDS) values. SDS values were calculated using LMS method and were based on normative ABPM data published by Wuehl et al. [25]. The change of measured parameters over time was assessed by delta value expressed as the difference between second and first value. BMI and WC were expressed in absolute values and as SDS based on Polish normative values for age and sex [26]. Similarly, cIMT and PWV values were analyzed as SDS.

Categorical variables before and after treatment were compared using the McNemar test, categorical variables between two independent groups (change in cSBP and change from sHT to tHT status) were compared using Chi-square test. All continuous variables were checked for normal distribution using the Shapiro-Wilk test. Normally distributed variables are presented as mean ± SD; non-normally distributed variables are shown as medians with interquartile ranges. For pairwise repeated measure comparisons of parameters before and after treatment, we used paired *t* test (normally distributed variables) or paired Wilcoxon test (for non-normally distributed variables).

Independent measures of two subgroups were compared using an unpaired *t* test (normally distributed variables); non-parametric variables were presented as medians and IQR (Tables 2 and 3), but their distribution was normalized by using cube root (power to 1/3) transformation (in order to transform negative and positive data values), and all variables were subsequently compared with a parametric unpaired *t* test. This allowed us to calculate and compare Cohen *d* effect size (expressed as SD) of all (parametric and non-parametric variables), which, unlike significance tests, is independent of sample size. Cohen *d* ≤ 0.2 can be considered a “small” effect size, 0.5 represents a “medium” effect size, and 0.8 a “large” effect size [27]. Three variables with the highest Cohen effect size were then included into the multiple regression analysis in order to assess determinants of cSBP change (increase/decrease) over time.

The threshold of a significant *p* value (usually < 0.05) was adjusted for multiple testing using Bonferroni correction, by dividing the obtained *p* values by the number of performed tests (indicated below each table). Statistics were performed using Python (version 3.7.0., packages TableOne and Pingouin) [28].

## Results

After 12 ± 3 months, patients’ height increased, whereas BMI-SDS decreased. There were no significant changes during the observation period in biochemical and TOD parameters and the 24-h SBP, DBP, and MAP decreased over time (Table 1, Table 1S).

**Table 1:** Characteristics of patients group (paired comparison) at the baseline and after 12 months

Parameters	Baseline (*n* = 43)	After 12 months (*n* = 43)	*p*
Age (years)	16.7 (16.0;17.0)	17.5 (16.9;17.9)	0.00001
Weight (kg)	72.0 (62.7; 81.8)	74.2 (66.2; 82.2)	0.4
Height (cm)	176.0 (169.3; 181.3)	178.0 (169.8; 183.0)	0.00001
BMI (kg/m^2^)	23.5 (21.9; 26.3)	23.0 (22.2; 25.6)	0.2
BMI-SDS	0.95 ± 0.90	0.79 ± 0.88	0.002
WC (cm)	78.3 (75.0; 84.6)	79.0 (75.5; 84.5)	0.5
WC-SDS	0.90 ± 0.96	0.85 ± 0.98	0.05
SBP (mmHg)	129 ± 9	130 ± 9	0.4
DBP (mmHg)	68 ± 8	68 ± 6	0.3
pPP (mmHg)	61 ± 7	62 ± 8	0.6
24-h SBP (mmHg)	134 ± 6	131 ± 7	0.0006
24-h DBP (mmHg)	75 (71;76)	73 (69;75)	0.2
24-h SBPi	1.03 (1.02; 1.05)	1.01 (0.98; 1.04)	0.0003
24-h DBPi	0.94 (0.89; 0.96)	0.92 (0.87; 0.95)	0.1
24-h MAP (mmHg)	94 ± 5	90 ± 5	0.00001
24-h MAP-SDS	1.61 ± 0.86	0.90 ± 0.93	0.00001
PPA (mmHg)	14 (11;160	13 (10;17)	0.1
cSBP (mmHg)	116 (111;120)	120 (116;123)	0.0000
cSBPi	0.97 (0.94; 0.98)	0.98 (0.95;1.01)	0.03
AugPress (mmHg)	3 (2;5)	3 (2;5)	0.5
AugInd	6 (4;10)	7 (5;10)	0.2
HR (`/min)	76 ± 11	74 ± 9	0.04
SV (ml)	83 ± 17	85 ± 16	0.2
CO (l/min)	6.0 ± 1.5	5.8 ± 1.4	0.3
CI (l/min/m^2^)	3.2 (2.6; 3.7)	3.0 (2.5; 3.5)	0.1
TPR (PRU)	0.91 (0.77; 1.09)	0.91 (0.81; 1.10)	0.5
PWV (m/s)	5.8 ± 0.6	5.8 ± 0.5	0.1
PWV SDS	1.55 ± 1.0	1.15 ± 1.08	0.005
PWV SDS ≥ 1.88	17/43	9/43	0.07
cIMT (mm)	0.45 (0.43; 0.47)	0.45 (0.44; 0.47)	0.2
cIMT SDS	1.0 (0.48; 1.59)	1.04 (0.72; 1.52)	0.8
CIMT SDS ≥ 1.65	7/43	10/43	0.7
WCSA (mm^2^)	7.4 (6.9; 7.8)	7.4 (6.9; 7.9)	0.8
WCSA-SDS	0.75 (0.31; 1.3)	0.78 (0.36; 1.25)	0.4
LVMi (g/m^2.7^)	35.0 ± 4.7	34.0 ± 5.6	0.2
LVH (LVMi ≥ 95c)	7/43	7/43	0.7

Normally distributed variables shown as mean and SD, compared with paired *t* test

Non-normally distributed variables shown as median and interquartile range, compared with Wilcoxon rank-sum test

Categorical variables compared with McNemar test. Unadjusted *p* values shown. Adjustment for multiple testing using Bonferroni correction: adjusted threshold for statistical significance of 0.05/number of tests (*n* = 37) = 0.0013

*BMI*, body mass index; *WC*, waist circumference; *SBP*, systolic blood pressure; *DBP*, diastolic blood pressure; *pPP*, peripheral pulse pressure; *24-h SBP*, 24-h systolic blood pressure; *24-h SBPi*, 24-h systolic blood pressure index (24-h SBP/95 percentile for height and sex); *24-h DBP*, 24-h diastolic blood pressure; *24-h DBPi*, 24-h diastolic blood pressure index (24-h DBP/95 percentile for height and sex); *24-h MAP*, 24-h mean arterial pressure; *PPA*, pulse pressure amplification; *cSBP*, central systolic blood pressure; *cSBPi*, central systolic blood pressure index (cSBP/95 percentile for age and sex); *AugPress*, augmentation pressure; *AugInd*, augmentation index; *HR*, heart rate; *SV*, stroke volume; *CO*, cardiac output; *CI*, cardiac index; *TPR*, total peripheral resistance; *PWV*, pulse wave velocity; *cIMT*, carotid intima-media thickness; *WCSA*, wall cross-sectional area; *LVMi*, left ventricular mass index; *LVH*, left ventricular hypertrophy

**Fig. 2:**
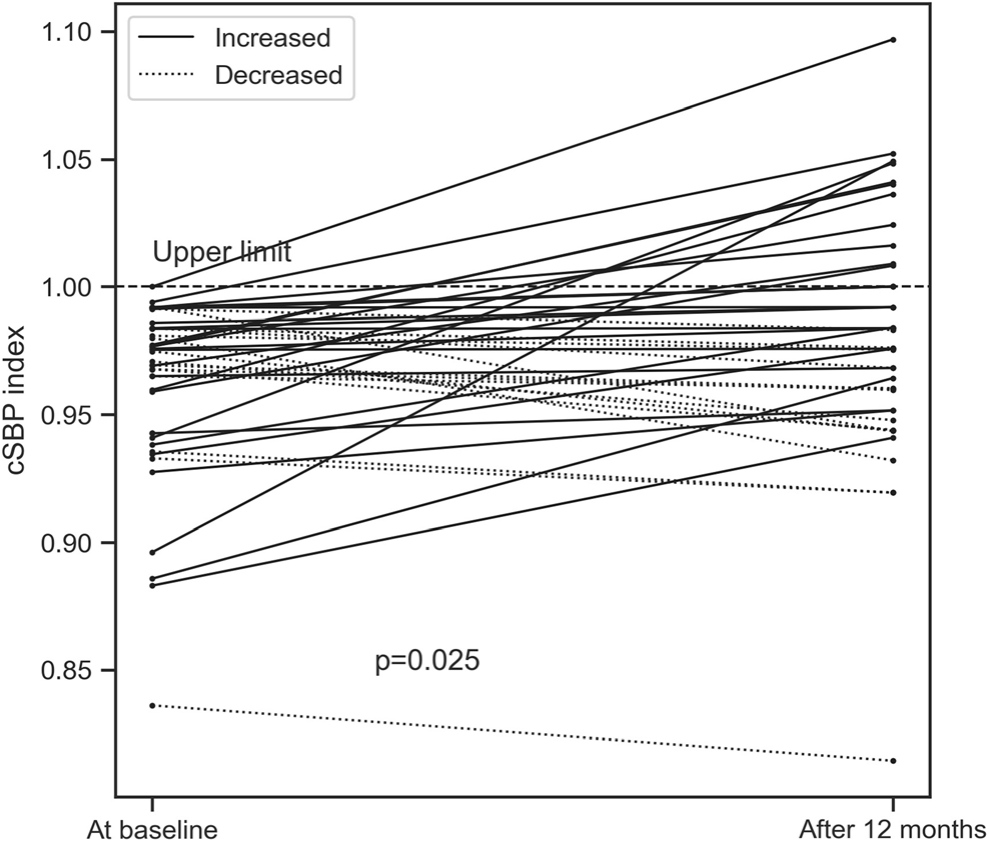
Change of central systolic blood pressure index (cSBP index) at the baseline and after 12 months. cSBPi, central systolic blood pressure/95th percentile for age and sex

**Fig. 3:**
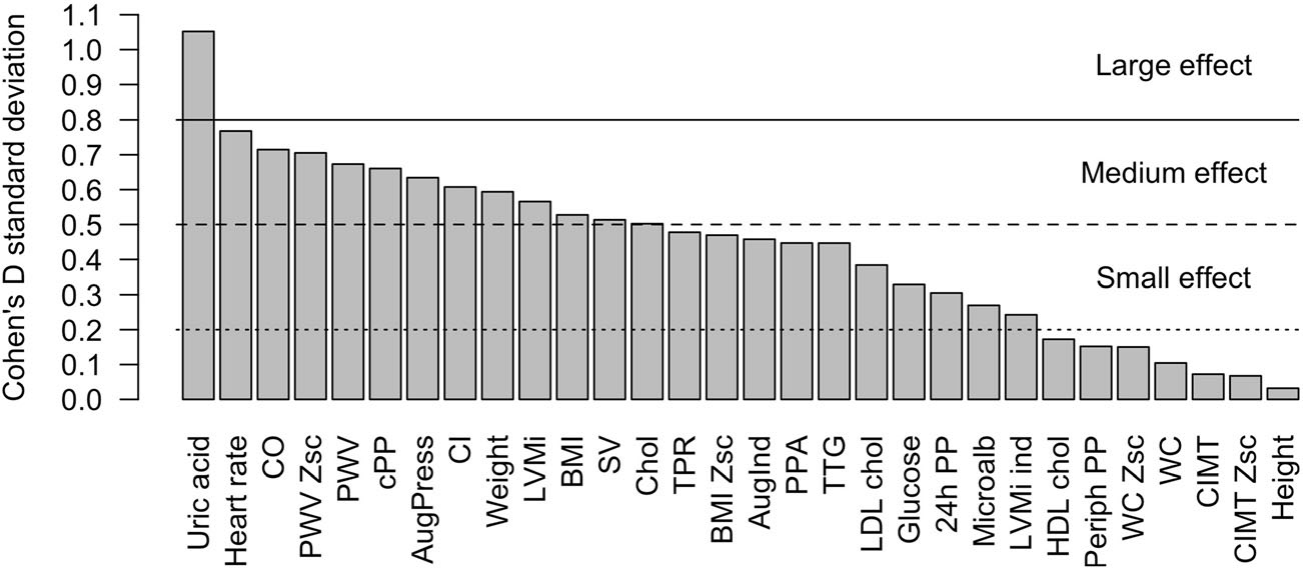
Effect size of variables on the change of sHT to tHT class. sHT, spurious hypertension; tHT, true hypertension

Twenty-seven patients (63%) increased their cSBP over study period, 12 (28%) patients had an elevated cSBP > 1.0 after 12 months of follow-up (Fig. 2), but the median cSBP was still below the upper limit of normal at baseline and after 12 months (Table 1). Patients who increased their cSBP (*n* = 27) also increased heart rate, cardiac output, PWV SDS, cholesterol and serum uric acid concentrations (Table 2). The change of uric acid concentration was associated with the highest/large effect size (Cohen *d* = 1.05) on the change of cSBP, the change in following variables had a medium to large effect size (Cohen *d* = 0.5 to 0.8) on the change of cSBP: heart rate, cardiac output, PWV SDS, PWV, cPP, AugPress, cardiac index, weight, LVMi, BMI, stroke volume, and cholesterol (Fig. 3). Moreover, there was significant correlation between change of uric acid serum levels and change of heart rate (*r* = 0.030; *p* = 0.04, Spearman test).

**Table 2:** Anthropometric, biochemical, hemodynamic, and TOD parameter differences in groups by central systolic blood pressure index (cSBPi) change (Cohen’s effect size). Values expressed as mean and standard deviation or median and interquartile range, where appropriate. Unadjusted *p* values are shown. Adjustment for multiple testing using Bonferroni correction: adjusted threshold for statistical significance of 0.05/number of tests (*n* = 37) = 0.0013

Parameters	cSBPi decreased/unchanged (*n* = 16)	cSBPi increased (*n* = 27)	*p*	Effect size
Δ Weight (kg)	− 1.2 (− 3.4; 1.5)	0.6 (− 0.7; 2.6)	0.09	0.594
Δ Height (cm)	1.4 (0.7; 2.3)	1.0 (0.5; 2.8)	0.414	0.032
Δ BMI (kg/m^2^)	− 0.7 ± 1.3	0 ± 1.4	0.107	0.528
Δ BMI-SDS	− 0.26 ± 0.32	− 0.11 ± 0.31	0.147	0.469
Δ WC (cm)	− 0.8 ± 2.9	− 0.4 ± 3.2	0.819	0.105
Δ WC-SDS	− 0.17 ± 0.35	− 0.12 ± 0.32	0.735	0.15
Δ SBP (mmHg)	− 4 ± 5	5 ± 8	0.001	1.307
Δ DBP (mmHg)	− 1 ± 5	3 ± 6	0.055	0.673
Δ pPP (mmHg)	− 2 (− 5; 3)	− 2 (− 5; 1)	0.386	0.447
Δ 24-h SBP (mmHg)	− 5 ± 4	− 3 ± 7	0.373	0.322
Δ 24-h DBP (mmHg)	− 3 ± 3	0 ± 5	0.064	0.655
Δ 24-h SBPi	− 0.04 ± 0.03	− 0.03 ± 0.05	0.455	0.274
Δ 24-h DBPi	− 0.03 ± 0.04	0 ± .06	0.1	0.579
Δ 24-h MAP (mmHg)	− 6 ± 4	− 3 ± 6	0.122	0.545
Δ 24-h MAP-sds	− 1.03 ± 0.68	− 0.59 ± 1.03	0.16	0.504
Δ PPA (mmHg)	− 2 (− 5; 3)	− 2 (− 5; 1)	0.386	0.447
Δ cSBP (mmHg)	0 (− 2; 1)	6 (2; 11)	< 0.001	1.603
Δ cSBPi	− 0.01 (− 0.02; − 0.01)	0.04 (0.01; 0.06)	< 0.001	1.603
Δ AugPress (mmHg)	0 ± 2	1 ± 2	0.067	0.634
Δ AugInd	0 ± 3	1 ± 3	0.179	0.458
Δ HR (`/min)	− 5 ± 5	0 ± 7	0.028	0.768
Δ SV (ml)	− 4 (− 9; 4)	5 (− 1; 7)	0.094	0.513
Δ CO (l/min)	− 0.6 ± 1.0	0.1 ± 1.0	0.041	0.715
Δ CI (l/min/m^2^)	− 0.4 ± 0.7	− 0.1 ± 0.7	0.077	0.608
Δ TPR (PRU)	0.09 ± 0.18	0 ± 0.21	0.163	0.478
Δ PWV (m/s)	− 0.3 ± 0.3	0 ± 0.4	0.056	0.673
Δ PWV z-score	− 0.76 ± 0.77	− 0.19 ± 0.84	0.038	0.705
Δ cIMT (mm)	0 (− 0.01; 0.02)	0 (− 0.01; 0.02)	0.897	0.072
Δ cIMT z-score	− 0.17 (− 0.31; 0.36)	− 0.01 (− 0.31; 0.38)	0.856	0.067
Δ LVMi (g/m^2.7^)	0.80 ± 4.8	− 2 ± 5	0.082	0.566
Δ Glucose (mg/dl)	1 ± 5	− 1 ± 6	0.422	0.328
Δ Uric acid (mg/dl)	− 0.4 (− 2.1; − 0.1)	- 0.1 (− 0.3; 0.3)	0.028	1.052
Δ Total cholesterol (mg/dl)	− 13 ± 20	0 ± 30	0.028	0.502
Δ LDL cholesterol (mg/dl)	− 9 ± 18	− 2 ± 19	0.293	0.384
Δ HDL cholesterol (mg/dl)	− 1 (− 3; 6)	1 (− 2; 4)	0.985	0.173
Δ Triglycerides (mg/dl)	− 5 ± 30	9 ± 31	0.226	0.447
Δ Microalbumin excretion (mg/24 h)	0.6 (− 2.9; 1.3)	− 1.8 (− 7.2; 3.9)	0.846	0.27

*BMI*, body mass index; *WC*, waist circumference; *SBP*, systolic blood pressure; *DBP*, diastolic blood pressure; *pPP*, peripheral pulse pressure; *24-h SBP*, 24-h systolic blood pressure; *24-h SBPi*, 24-h systolic blood pressure index (24-h SBP/95 percentile for height and sex); *24-h DBP*, 24-h diastolic blood pressure; *24-h DBPi*, 24-h diastolic blood pressure index (24-h DBP/95 percentile for height and sex); *24-h MAP*, 24-h mean arterial pressure; *PPA*, pulse pressure amplification; *cSBP*, central systolic blood pressure; *cSBPi*, central systolic blood pressure index (cSBP/95 percentile for age and sex); *AugPress*, augmentation pressure; *AugInd*, augmentation index; *HR*, heart rate; *SV*, stroke volume; *CO*, cardiac output; *CI*, cardiac index; *TPR*, total peripheral resistance; *PWV*, pulse wave velocity; *cIMT*, carotid intima-media thickness; *WCSA*, wall cross-sectional area; *LVMi*, left ventricular mass index; *LVH*, left ventricular hypertrophy

Ten of 43 patients (23.2%) developed tHT with elevated 24-h ABPM and cSBP values after 12 months, 22 evolved to WCH or normotension, and 11 maintained their status of sHT.

Comparison of baseline characteristics of 10 patients who developed tHT with another 33 patients who had maintained their cSBP or evolved to WCH or normotension, showed that patients who developed tHT significantly increased their office and 24-h ABPM values, and CO and UA levels (Table 3). While the statistical significance of change of other parameters relative to the change of sHT status was borderline, the change of CO and UA was associated with a large effect size (Cohen *d* > 0.8), whereas the effect size of the change of 24-h, peripheral and central pulse pressure, HDL-cholesterol, triglycerides, PWV, and cardiac index was medium to large (Cohen *d* = 0.5–0.8) (Table 3). Differences in sex distribution between groups did not attain statistical significance. However, numerically there were only 9% of females in the group which maintained or normalized their blood pressure status as compared with 30% in the group which progressed to tHT.

**Table 3:** Anthropometric, biochemical, hemodynamic, and TOD parameter changes in groups by blood pressure status change (Cohen’s effect size). Values expressed as mean and SD or median and interquartile range, where appropriate. Unadjusted *p* values are shown. Adjustment for multiple testing using Bonferroni correction: adjusted threshold for statistical significance of 0.05/number of tests (*n* = 37) = 0.0013

Parameters	sHT - > WCH/AmbPreHT/sHT (33)	sHT - > tHT (10)	*p*	Effect size
Δ Weight (kg)	0 (− 2; 1.6)	1.1 (0.5; 3.3)	0.191	0.49
Δ Height (cm)	1.1 (0.6; 2.0)	1.0 (0.7; 2.9)	0.886	0.322
Δ BMI (kg/m^2^)	− 0.3 ± 1.5	0 ± 1.0	0.576	0.231
Δ BMI-SDS	− 0.19 ± 0.34	− 0.08 ± 0.21	0.391	0.37
Δ WC (cm)	− 0.3 ± 2.7	− 1.3 ± 4.1	0.52	0.28
Δ WC-SDS	− 0.13 ± 0.35	− 0.18 ± 0.27	0.734	0.175
Δ SBP (mmHg)	− 1 ± 7	9 ± 8	0.001	1.402
Δ DBP (mmHg)	0 ± 6	5 ± 7	0.036	0.792
Δ pPP (mmHg)	0 (− 3; 3)	4 (1; 6)	0.074	0.642
Δ 24-h SBP (mmHg)	− 5 ± 5	1 ± 7	0.005	1.009
Δ 24-h DBP (mmHg)	− 2 ± 4	3 ± 5	< 0.001	1.321
Δ 24-h SBPi	− 0.04 ± 0.04	0 ± 0.05	0.009	0.962
Δ 24-h DBPi	− 0.04 ± 0.04	0.03 ± 0.06	0.001	1.214
Δ 24-h MAP (mmHg)	− 6 ± 4	1 ± 6	0.001	1.178
Δ 24-h MAP-sds	− 0.95 ± 0.69	− 0.01 ± 1.32	0.006	0.894
Δ PPA (mmHg)	− 2 (− 5; 2)	0 (− 5; 1)	0.791	0.042
Δ cSBP (mmHg)	1 (0; 3)	10 (8; 13)	< 0.001	2.044
Δ cSBPi	0 (− 0.01; 0.01)	0.06 (0.05; 0.07)	< 0.001	1.766
Δ AugPress (mmHg)	0 ± 2	1 ± 3	0.213	0.415
Δ AugInd	1 ± 3	1 ± 5	0.649	0.149
Δ HR (`/min)	− 2 ± 7	− 2 ± 6	0.958	0.021
Δ SV (ml)	2 (− 4; 6)	6 (− 1; 15)	0.289	0.417
Δ CO (l/min)	− 0.4 ± 0.9	0.5 ± 1.2	0.028	0.839
Δ CI (l/min/m^2^)	− 0.3 ± 0.6	0.1 ± 0.9	0.147	0.512
Δ TPR (PRU)	0.03 ± 0.17	0.07 ± 0.29	0.593	0.175
Δ PWV (m/s)	− 0.2 ± 0.3	0.1 ± 0.6	0.084	0.552
Δ PWV z-score	− 0.51 ± 0.77	− 0.08 ± 1.06	0.169	0.464
Δ cIMT (mm)	0.01 (− 0.01; 0.02)	− 0.01 (− 0.01; 0)	0.273	0.093
Δ cIMT z-score	0.10 (− 0.27; 0.38)	− 0.26 (− 0.38; − 0.12)	0.194	0.127
Δ LVMi (g/m^2.7^)	− 0.7 ± 4.5	− 1.8 ± 6.6	0.536	0.202
Δ Glucose (mg/dl)	0 ± 5	− 3 ± 6	0.257	0.489
Δ Uric acid (mg/dl)	− 0.5 ± 0.9	0.2 ± 0.5	0.018	0.871
Δ Total cholesterol (mg/dl)	− 8 ± 21	2 ± 43	0.404	0.28
Δ LDL cholesterol (mg/dl)	− 6 ± 16	− 2 ± 27	0.58	0.194
Δ HDL cholesterol (mg/dl)	3 ± 9	− 3 ± 7	0.088	0.655
Δ Triglycerides (mg/dl)	− 1 ± 29	18 ± 32	0.139	0.632
Δ Microalbumin excretion (mg/24 h)	0.6 (− 4.6; 2.5)	− 2.4 (− 11.4; 4.8)	0.734	0.487

*sHT*, spurious hypertension; *tHT*, true hypertension; *BMI*, body mass index; *WC*, waist circumference; *SBP*, systolic blood pressure; *DBP*, diastolic blood pressure; *pPP*, peripheral pulse pressure; *24-h SBP*, 24-h systolic blood pressure; *24-h SBPi*, 24-h systolic blood pressure index (24-h SBP/95 percentile for height and sex); *24-h DBP*, 24-h diastolic blood pressure; *24-h DBPi*, 24-h diastolic blood pressure index (24-h DBP/95 percentile for height and sex); *24-h MAP*, 24-h mean arterial pressure; *PPA*, pulse pressure amplification; *cSBP*, central systolic blood pressure; *cSBPi*, central systolic blood pressure index (cSBP/95 percentile for age and sex); *AugPress*, augmentation pressure; *AugInd*, augmentation index; *HR*, heart rate; *SV*, stroke volume; *CO*, cardiac output; *CI*, cardiac index; *TPR*, total peripheral resistance; *PWV*, pulse wave velocity; *cIMT*, carotid intima-media thickness; *WCSA*, wall cross-sectional area; *LVMi*, left ventricular mass index; *LVH*, left ventricular hypertrophy

In the multiple regression analysis we did not analyze BP-related parameters (SBP, DBP, MAP), since cSBP is derived from measurements of peripheral BP; i.e., the inherent relationship between peripheral and central BP may represent a potential bias in the prediction of cSBP. The multivariate regression analysis of the top three non-BP-related parameters with the highest effect size on univariate analysis (UA, HR, and CO, Fig. 3), showed that only the change of serum UA concentrations was a significant predictor (*β* = 0.225, 95% CI = 0.018–0.432, *p* = 0.03) of the change of cSBP.

### Target organ damage

At baseline, 7 of 43 (16%) patients had mildly increased cIMT (above 95th percentile), arterial stiffness (assessed as PWV) was increased (> 97th percentile) in 17 of 43 (40%) patients and elevated LVMi (> 95th percentile) indicating mild LVH (LVMi < 51 g/m height^2.7^) in 7 of 43 (16%) patients (Table 1). After 12 months, increased cIMT was noted in 10 patients (23%), increased PWV in 9 patients (21%) and LVH in 7 patients (16%), but all LVMi values were below 51 g/m height^2.7^. There were no significant differences in the change of hypertensive TOD parameters between groups which maintained sHT/normalized peripheral and central BP and those who progressed to tHT (Table 2; supplementary material).

## Discussion

The main finding of our study is that 23% of adolescents with ISH and normal cSBP who were prescribed only non-pharmacological therapy developed tHT after 12 months. The other group of 33 (77%) patients maintained (11 subjects) their sHT status or normalized peripheral and central BP (22 subjects). The main determinant of change in BP status was the change in serum UA concentration. These findings suggest that ISH and normal cSBP in adolescents is a heterogenous condition, and in a significant proportion of subjects is associated with the risk of development of sustained hypertension and hypertensive TOD, thus requiring close monitoring. These are novel findings, based on analysis of both peripheral and central blood pressure, and to our knowledge, this is the first prospective pediatric study which includes only adolescents and not adults, and included the greatest number of sHT cases described so far.

Starting from the first report of O’Rourke et al., several studies on ISH with normal cSBP were conducted in so-called young adults and included both adolescents and adults [1]. Mahmud and Feely assessed the prevalence of this phenomenon in a group of 174 medical students (aged 23 ± 0.5 years; 87 women) and 22 young hypertensive male adults aged 24.8 ± 0.88 years. ISH with normal cSBP was diagnosed in only 11 out of 174 (6.3%) of otherwise healthy normotensive students (all males) [29]. This is a lower prevalence of this phenotype of BP than in our previous cross-sectional study, in which we found ISH with normal cSBP in 35% of adolescents from the group of consecutive patients referred because of PH. Moreover, in our study, ISH was confirmed by office and ABPM. Mahmoud et al. provided data on 6/11 patients with sHT after 2 years of follow-up. All 6 patients maintained their status of ISH with normal cSBP, but only 2 patients had echocardiography performed showing no LVH [29].

The finding that 23% of subjects in our study increased their cSBP, i.e., became true hypertensive, is higher than in prospective studies of young adults, in whom the risk of developing sustained hypertension needing treatment was rather low (15.2%) and similar to the control group (14.7%) [3]. In both adolescents in the current study and 33 young adults with mean age of 33 years in the study of Saladini et al. [3], most subjects were males (86% vs. 94%, respectively). Because of the low number of subjects in each group, we did not find any statistically significant differences in sex distribution between those who maintained or normalized their BP status and those who increased cSBP, but numerically there were more females in the group which increased cSBP (9 vs. 30%, respectively). The 3 times higher percentage of females in the group which increased cSBP is in agreement with both our previous study (24.4%) and the overall sex distribution among adolescents with PH [9].

Saladini et al. found that among young adults with ISH and normal cSBP the long-term risk of developing sustained hypertension and needing treatment seems to be directly related to the level of cSBP, as 50% of patients with cSBP above normal at baseline developed sustained hypertension (stage 2) as compared with only 15.2% of patients with a lower cSBP at baseline [3]. Our 43 patients had similar baseline absolute values of cSBP (median = 116 mmHg) compared with patients from the Saladini et al. study (113.8 mmHg) [3]. However, median cSBP at the end of a 1 year follow-up was 120 mmHg (median increase by 4 mmHg per year). More importantly, the increase of cSBP was significantly higher in patients who developed tHT (median change = 11 mmHg/year) compared with patients who developed WCH or ambulatory prehypertension or those who maintained sHT (median change = 1 mmHg/year) (Table 2S). It may be hypothesized that over a longer period of time (10 years as in the study by Saladini et al.), some of our patients, mainly those who increased their cSBPi over 1 year (63%), would develop sustained hypertension. In addition, our patients were younger by almost 18 years, which suggests that earlier elevation of brachial blood pressure even with normal cSBP is associated with earlier development of sustained hypertension. However, only long-term observation time can prove this hypothesis.

Mild TOD, mainly in the form of increased PWV was found in 7 of 43 (16%) patients at study start. As reported previously, patients with ISH and normal cSBP had lower prevalence of LVH and lower values of cIMT and PWV than those with elevated cSBP [9]. The finding of mild forms of TOD in subjects with brachial hypertension and normal cSBP is not surprising because cIMT and LVMi increase with change of blood pressure status from normotension to severe ambulatory hypertension and cSBP was found to be one of the predictors of LVMi [30]. Although subjects included in our study had normal cSBP values, they were above median values [17].

The main determinant of change of cSBP over time was the change of serum UA concentrations. The role of UA in the pathogenesis of PH is well described and elevated serum UA levels are typical for adolescents with PH [31, 32]. Moreover, the decrease of UA during treatment with allopurinol also lowered BP [32]. Previously we found that a change in UA was one of the predictors of regression of hypertensive TOD [33]. There are no reports on the association between serum UA and cSBP in hypertensive children. However, serum UA was found to be associated with the increased expression of *MMP-14* in peripheral blood leucocytes of hypertensive children [34]. This may be a potential explanation for the link between an increase in UA and cSBP over time, because *MMP-14* is involved in arterial remodeling and enhances *MMP-2* activity, which has been found to be the most significant marker of matrix metalloproteinases/tissue inhibitors of the matrix metalloproteinase system activation in hypertensive children [34, 35]. Increased serum UA can also be associated with adrenergic drive [36]. The association of increased serum UA with an increased HR and CO, along with an increase of cSBP, suggests the role of sympathetic drive in the evolution from sHT to tHT. An association between CO and cSBP was also found in a recent study of hemodynamics in adolescents with PH [37].

In contrast to our previous studies we did not find any changes in BMI or WC, both expressed in absolute and standardized values, as predictors of cSBP change. However, it must be underlined that subjects from this study had relatively normal BMI and WC values which were close to the median of the population norm (BMI-SDS and WC-SDS both below 1.0) and had lower BMI and WC than patients with ISH and elevated cSBP, as reported previously [9].

According to O’Rourke’s hypothesis [1], ISH with normal cSBP in adolescent and young adult males is caused by an increased elasticity of middle-sized arteries, such as the brachial artery, when compared with adults. It allows for the accumulation of a backward pulse wave via the brachial artery with a rise in brachial SBP but cSBP and cPP do not change. This phenomenon occurs more often in boys and in taller adolescents. It is in contrast to ISH in elderly, where a rise of SBP is caused by increased stiffness of the arterial tree and faster return of the backward wave to the aorta. In our study, of all the patients studied, 41/48 (85%) were males. There were also more males (91% vs. 70%) in the group of patients who after observation maintained sHT or normalized their BP status. This group was also taller (177 cm vs. 169 cm); however, this difference is not significant and likely due to the limited number of study participants (Table 1).

Our study has several limitations. Firstly, although we prospectively analyzed the greatest number of patients with sHT reported so far, the number of study subjects was relatively low. Moreover, the role of sex was difficult to analyze due to the low number of females. Secondly, the observation period was limited to 1 year, which may not be long enough to develop any significant changes in hemodynamic and TOD parameters. However, as changes in the ABPM blood pressure status in adolescents are described [38], cSBP change over time is also to be expected. The other limitation is that we did not objectively assess adherence to non-pharmacological therapy. However, both mean BMI-SDS and WC-SDS values decreased after 12 months, which suggests adherence to non-pharmacological treatment.

The strength of our study stems from the fact that it is the first prospective pediatric study to include an unselected group of adolescents who were referred for work-up of PH and in whom a comprehensive assessment of hemodynamic parameters including ABPM, cSBP, TOD assessment, and laboratory investigations was done. Second, this is the largest prospective study to date analyzing the evolution of ISH with normal cSBP. Third, all subjects had all tests completed at baseline and after 1 year of follow-up. Fourth, although unselected and consecutively referred, our patients were relatively lean, non-obese, which allowed us to study the impact of factors not related to obesity.

## Conclusions

Our findings document that ISH with normal cSBP is not benign and may progress to elevated cSBP in a significant proportion (23%) of patients. The main determinant of change of cSBP over time was the change of serum UA concentration. Thus, our results suggest that adolescents with ISH and normal cSBP are a heterogenous group and that metabolic abnormalities play a significant role in the rise of cSBP, even in the cohort of non-obese hypertensive children. cSBP is not routinely measured in hypertensive children and adolescents, and management depends on hypertension severity and presence of TOD. Although there is a relationship between brachial blood pressure and cSBP—the higher the brachial blood pressure, the greater the probability of an elevated cSBP—almost one third of our patients with severe ambulatory hypertension had normal cSBP [9]. Thus, assessment of cSBP may be a useful tool in selected cases with ISH, without hypertensive TOD and without metabolic cardiovascular risk factors.

## Electronic supplementary material

ESM 1 (DOCX 14 kb)
