# Supplementary material for: Evolution of isolated systolic hypertension with normal central blood pressure in adolescents—prospective study
Source: Pediatr Nephrol. 2020 Sep 3;36(2):361–71. doi: 10.1007/s00467-020-04731-z (PMC7815547; doi:10.1007/s00467-020-04731-z)
Supplement: Supplementary file 1 — (DOCX 14 kb) [file 467_2020_4731_MOESM1_ESM.docx]

Supplementary material

*Patients normalizing blood pressure in ABPM or maintaining sHT after observation (sHT-NT/sHT-sHT)*

At baseline 4/33 patients in this group (12%; 2 girls) had increased cIMT (cIMT-SDS ≥1.65). 1 of them normalized cIMT during the observation.

In 4 out of 33 patients (1 girl; 12%), LVMi above 95^th^ percentile but still below 51g/m height^2.7^ was already diagnosed at the beginning of observation. After 12±3 months of non-pharmacological treatment, 2 patients normalized LVMi and 2 patients maintained their LVMi.

PWV was increased (PWV-SDS ≥1.88) in 14 patients in this group (3 girls; 42%) at baseline, however 7 of them normalized during non-pharmacological treatment

*Patients’ Developing tHT (sHT-tHT)*

In this subgroup 30% of patients (3/10; 2 girls) presented with increased cIMT at baseline and all of them maintained it during non-pharmacological treatment

LVMi above 95^th^ percentile was found in 30% of the patients (3/10; 1 girl) who developed tHT, all of which had their LVMi normalized during treatment

PWV was increased primarily in 3 patients (30%; boys) and in all cases normalized by the end of observation

Table 1S. Characteristic of patients group (paired comparison of biochemical parameters) at the baseline and after the observation.

| **Parameters** | **Baseline (n=43)** | **After 12 months (n=43)** | **p** |
| --- | --- | --- | --- |
| Glucose  [mg/dl] | 88±7 | 87±5 | 0.5 |
| Uric acid [mg/dl] | 5.9 ± 1.6 | 5.7 ± 1.1 | 0.07 |
| Total cholesterol [mg/dl] | 162 (145;186) | 163 (147;182) | 0.2 |
| LDL cholesterol [mg/dl] | 97±26 | 93±26 | 0.5 |
| HDL cholesterol [mg/dl] | 51 (44;62) | 52 (42;63) | 0.1 |
| Triglicerydes [mg/dl] | 93 (77;123) | 96 (81;133) | 0.5 |
| Microalbumin excretion [mg/24h] | 10.8 (4.7; 15.7) | 7.9 (6.2; 13.1) | 0.6 |
